# Ciprofloxacin and Clinafloxacin Antibodies for an Immunoassay of Quinolones: Quantitative Structure–Activity Analysis of Cross-Reactivities

**DOI:** 10.3390/ijms20020265

**Published:** 2019-01-11

**Authors:** Andrey A. Buglak, Ilya A. Shanin, Sergei A. Eremin, Hong-Tao Lei, Xiangmei Li, Anatoly V. Zherdev, Boris B. Dzantiev

**Affiliations:** 1 A. N. Bach Institute of Biochemistry, Research Center of Biotechnology of the Russian Academy of Sciences, 33 Leninsky Prospect, 119071 Moscow, Russia; zherdev@inbi.ras.ru (A.V.Z.); dzantiev@inbi.ras.ru (B.B.D.); 2 Faculty of Physics, St. Petersburg State University, 7/9 Universitetskaya nab., 199034 St. Petersburg, Russia; 3 Chemical Department, M. V. Lomonosov Moscow State University, Leninskie Gory, 119991 Moscow, Russia; numenor-08@mail.ruf (I.A.S.); eremin_sergei@hotmail.com (S.A.E.); 4 XEMA Company Limited, Ninth Parkovaya street 48, 105264 Moscow, Russia; 5 Guangdong Provincial Key Laboratory of Food Quality and Safety, South China Agricultural University, Guangzhou 510642, China; hongtao@scau.edu.cn (H.-T.L.); lixiangmei12@163.com (X.L.)

**Keywords:** polyclonal antibodies, fluoroquinolones, immunoassay, quantitative structure-activity relationship analysis, ciprofloxacin, clinafloxacin

## Abstract

A common problem in the immunodetection of structurally close compounds is understanding the regularities of immune recognition, and elucidating the basic structural elements that provide it. Correct identification of these elements would allow for select immunogens to obtain antibodies with either wide specificity to different representatives of a given chemical class (for class-specific immunoassays), or narrow specificity to a unique compound (mono-specific immunoassays). Fluoroquinolones (FQs; antibiotic contaminants of animal-derived foods) are of particular interest for such research. We studied the structural basis of immune recognition of FQs by antibodies against ciprofloxacin (CIP) and clinafloxacin (CLI) as the immunizing hapten. CIP and CLI possess the same cyclopropyl substituents at the N1 position, while their substituents at C7 and C8 are different. Anti-CIP antibodies were specific to 22 of 24 FQs, while anti-CLI antibodies were specific to 11 of 26 FQs. The molecular size was critical for the binding between the FQs and the anti-CIP antibody. The presence of the cyclopropyl ring at the N1 position was important for the recognition between fluoroquinolones and the anti-CLI antibody. The anti-CIP quantitative structure–activity relationship (QSAR) model was well-equipped to predict the test set (*pred_R*^2^ = 0.944). The statistical parameters of the anti-CLI model were also high (*R*^2^ = 0.885, *q*^2^ = 0.864). Thus, the obtained QSAR models yielded sufficient correlation coefficients, internal stability, and predictive ability. This work broadens our knowledge of the molecular mechanisms of FQs’ interaction with antibodies, and it will contribute to the further development of antibiotic immunoassays.

## 1. Introduction

**Figure 1:**
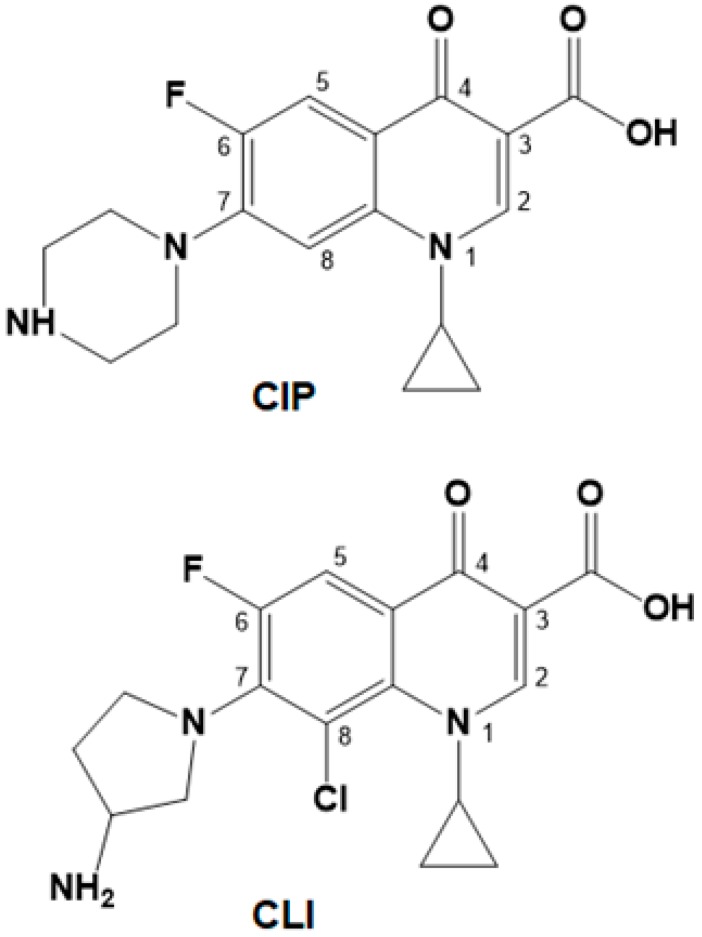
Molecular structure and atom numbering for ciprofloxacin (CIP) and clinafloxacin (CLI).

Fluoroquinolones (FQs) are a class of widely used antibiotic compounds [1]. The fluoroquinolone structure is based on a quinoline ring system. Carboxyl and fluorine are attached to the C3 and C6 positions, respectively, while carbonyl is located at the C4 position of the quinoline (Figure 1). The variation of four radicals (at the N1, C5, C7 and C8 position) determines the diversity of fluoroquinolone molecules.

Fluoroquinolones are effective against most gram-negative bacteria, as well as some Gram-positive bacteria, and for this reason, they are widely used in veterinary medicine; hence, foods of animal origin may be contaminated with fluoroquinolones [2]. In this way, bacterial resistance to FQs is induced and spread among human and animal pathogens—especially with *Campylobacter*, *E. coli*, and *Salmonella* [3,4,5,6]. In addition, low doses of FQs are transferred along food chains to humans, causing toxicological effects [7,8,9]. Actual data about these effects demonstrate that changes in the human microbiome are key contributors to further dysfunctions whose side effects extend to immune and metabolic diseases [10].

The risks associated with the consumption of FQs call for efficient techniques to control FQs in foods and environmental objects [11,12] as well as to monitor their levels during medical use [13]. Various instrumental techniques, including high performance liquid chromatography (HPLC), reversed phase high performance liquid chromatography (RP-HPLC), capillary electrophoresis (CE), UV-vis and fluorescent spectroscopy, have been developed for FQ control [14,15,16,17]. They are sensitive and accurate techniques; however, they are time-consuming, laborious, and have low throughput. On the contrary, immunoassays relying on antigen–antibody interactions are low-cost, have high throughput, and are easily automated. Therefore, their applications for the control of toxic food contaminants is a promising direction for modern developments [18,19]. A row of techniques has been proposed for immunodetection of fluoroquinolones in different food matrixes (including enzyme-linked immunosorbent assays (ELISAs), lateral flow immunoassays (LFIAs), and different immunosensors), and introduced to practice as commercial ELISA and LFIA kits—see recent review [20].

However, the development and application of immunoanalytical techniques require a clear understanding of how immunoassays recognize and distinguishes structurally close molecules. Different practical tasks in the control of toxic food contaminants demand either simultaneous determination of the compounds belonging to the same chemical class, or the ability to distinguish a limited row of compounds from their structural analogs [21,22]. In this line, several studies have presented immunotechniques for FQs’ detection with broad specificity [23,24,25,26,27,28,29,30]. However, choosing the best immunogen and competing derivative of FQ (conjugated with a protein or fluorescent tracer) is still empirical. The development of immunotechniques for the selective recognition of one or a few FQs is presented in several other studies [31,32,33,34,35,36], but it lacks the theoretical background to identify the unique immunogenic structures of specific FQs. Thus, an efficient further development of immunoassay protocols for FQs substantially requires new knowledge about the fundamental structural regularities of immune recognition.

Quantitative regulations of immune recognition were recently formulated for monoclonal antibodies against ciprofloxacin (CIP) [23]. A potential of sarafloxacin [29], pazufloxacin [37], marbofloxacin, benfloxacin, norfloxacin, and pefloxacin [37] for broad-specific detection of FQs was also demonstrated. Polyclonal antibodies against clinafloxacin (CLI) were obtained and used for the immunoassay of FQs in milk [38]. CLI possesses the same cyclopropyl substituent at the N1 position as CIP, while its substituents at C7 and C8 are different. In this context, the comparison of anti-CLI and anti-CIP antibodies with the use of the quantitative structure–activity relationship (QSAR) provides insight into how the differences of structurally related haptens influence fluoroquinolone recognition and immunoassay specificity.

Conformational analysis and QSAR analysis provide valuable information on the structural features of the quinolone haptens that affect the specificity of corresponding antibodies [24,26,37]. According to L. Cao and coauthors, substituents at positions 1 and 7 (for atom numbering, see Figure 1) are the most important for the selection of haptens, and the production of broad-specific antibodies against quinolones [24]. QSAR analysis of fluoroquinolones is widely used, mostly to predict fluoroquinolone antibacterial activity [39,40]. There are numerous examples of QSAR application for the study of the immunochemical recognition of fluoroquinolones [24,26,37,41,42]. Most of these studies employed 3D-QSAR analysis: comparative molecular field analysis (CoMFA), and/or comparative molecular similarity index analysis (CoMSIA). However, the internal stability and predictive ability of these models was quite restrained. The consideration of 3D-based parameters reflects the properties of native FQs, whereas their derivatives conjugated with protein or fluorescent tracers and used for immunization and competitive immunoassays may have some other conformations. So, we have considered the physical properties of chemical compounds (or their fragments), which are invariant with respect to compounds’ spatial orientations, as a basis to search for factors that determine the cross-reactivity of FQs in immunoassay. Moreover, 2D-QSAR was successfully used to predict fluoroquinolone antimicrobial activity with reasonable accuracy [40]. In this respect, we decided to use 2D-QSAR instead of 3D-QSAR in the given study.

Taking into account all the above facts, our aim was to raise antibodies against CIP and CLI haptens, and to define the molecular parameters that determine the recognition between these antibodies and quinolones. 2D-QSAR was used for the analysis of the recognition between the antibodies obtained and 26 quinolones.

## 2. Results

Two types of polyclonal antibodies were raised: an antibody against CIP and an antibody against CLI. The system of heterologous tracer PAZ-FITC (synthesized with pazufloxacin and FITC) and the antibody against ciprofloxacin (CIP-113/PAZ-FITC) recognized 22 of 24 tested quinolones (Table 1). Only sarafloxacin and difloxacin were not detected by anti-CIP antibodies, which coincides with the data previously reported by Wang et al. about the broad specificity of anti-CIP antibodies [23]. The CLI-132/CLI-С5-OVA system was specific to 11 of 26 quinolones: CLI, GAR, DAN, MOX, NAD, CIP, ENR, ENO, GAT, SPA, and ORB. However, cross-reactivity values were higher than those in the recent study by Chen et al. [36].

The multiple linear regression equations obtained for the studied systems are given below. Equation (1) (Model 1) represents the best-performing QSAR model for the activities of fluoroquinolones in the CIP-113/PAZ-FITC system.

Log CR = 4.338(±0.411) + 0.337(±0.048) × N(>CH-) − 0.075(±0.010) × Shadow_YZ(1)

N(>CH-) (relative contribution 36.86%), which is an amount of methantriyl (>CH-) groups. The N(>CH-) parameter is directly proportional to the activity, which means that the presence of >CH- groups is favorable for the activity. DAN, GAR, MOX, and similar analogues possess high N(>CH-) values and high activities. The values of N(>CH-) parameter are presented in Table 2.

**Figure 2:**
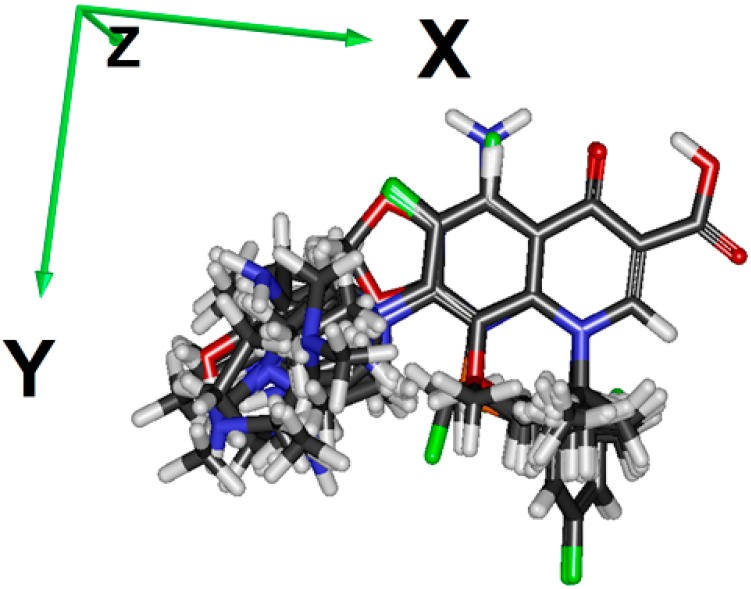
Coordinate axes and alignment of quinolone molecules.

Shadow-YZ (−63.14%) is an area of molecular shadow in the YZ plane (see Figure 2), and it has a reverse relation to the activity. It means that molecules with smaller projections on the YZ plane have higher activities (OXO, NAL, and similar analogues).

Equation (2) (Model 2) represents the best-performing QSAR model for the activity of fluoroquinolones in the CLI-132/CLI-С5-OVA, ELISA system:

Log CR = −0.225(±0.089) + 1.633(±0.130) × N(>CH-)+ 0.303(±0.091) × S(>CH-) − 1.749(±0.204) × N(Stereo)(2)

N(>CH-) (65%) is directly proportional to the activity in Model 2. It reveals that >CH- groups are favorable for the activity (DAN, GAR, MOX, and similar analogues).

N(Stereo) (−30.46%) is a number of stereo atoms, and it has a reverse relation to the activity. A small amount or the absence of stereo atoms is favorable for the activity (CIP, ENR, and similar analogues).

S(>CH-) (4.54%) represents a sum of the Kier–Hall electro-topological state indices [43] for carbons with three single bonds, and is directly proportional to the activity. Positive values of the electro-topological state of >CH- methantryil groups are favorable for the activity (DAN, MOX, and similar analogues).

**Figure 3:**
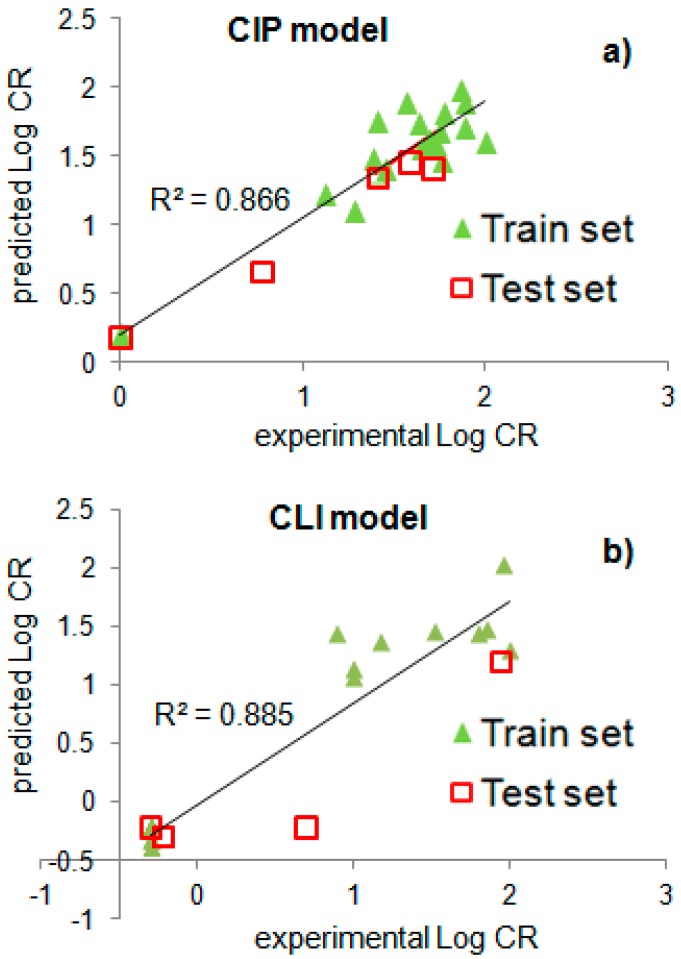
Graphs of experimental vs. predicted log CR values for fluoroquinolone molecules: (**a**) Model CIP/PAZ-FITC and (**b**) Model CLI/CLI-С5-OVA, ELISA.

The QSAR model is considered to be predictive if the following conditions are satisfied: *r_tr_*^2^ > 0.6, *q*^2^ > 0.5, and *pred_r*^2^ > 0.5. The statistical results generated by QSAR analysis show that both QSAR models have acceptable internal as well as external predictive abilities (Table 3). The results obtained for the actual and predicted cross-reactivities of different fluoroquinolones are presented in Figure 3 and in supplementary materials (Table S1).

## 3. Discussion

The statistical parameters of the obtained models (Table 3) are comparable, or they even surpass the analogous parameters of the previously reported 3D-QSAR models. The CoMFA model to recognize the interaction between the FQs and anti-ciprofloxacin antibodies was studied on 14 compounds with no external validation set [23]; we performed the analysis for 24 compounds and obtained a model with high predictive power toward the test set (*pred_r*^2^ = 0.944). The CoMFA model to recognize the interaction between FQs and anti-clinafloxacin antibodies is characterized by *q*^2^ = 0.587 [44], while our anti-CLI model had *q*^2^ = 0.864. Consequently, our 2D-QSAR analysis results are reasonable and useful on a par with 3D-QSAR data.

An interpretation of the results has led us to several observations. Equation (1) and the relative contribution of its variables to Model 1 show that quinolone recognition by the anti-CIP antibody essentially depends on the size of the molecule: molecules that are more compact in the YZ dimension possess higher activities. The smallest molecules among those considered, nalidixic acid (CR = 77%) and flumequine (CR = 72%), possess high activities, but only due to the availability of the quinolone structure and the absence of bulky substituents. FQs with bulky substituents at the N1, C7, and C8 positions (for atom numbering see Figure 1) should have lower recognition with anti-CIP antibodies. In particular, this means that molecules with a fluorophenyl ring attached to the N1 position (DIF, SAR, and TOZ) have low CR values (≤6) because the fluorophenyl ring attached to N1 is perpendicular to the quinolone ring system (for 3D geometries see Table S1) and enlarges molecule size in the YZ dimension. The addition of methyl and ethyl radicals to the piperazinyl substituent at the C7 position is also unfavorable for the activity. This observation coincides with the recent data by Chen et al. [44]. >CH- groups are also favorable for high cross-reactivity.

According to Model 2 (immunoassay based on anti-CLI antibodies), the presence of methantriyl groups is favorable for high cross-reactivity. However, not all methantriyl groups are of the same quality, and they increase quinolone activity. These methantriyl groups which refer to the stereo atoms of the C7 substituent (N(Stereo) parameter), have a negative influence on the value of cross-reactivity (for example, GAT and SPA). Electronegative atoms (S(>CH-) parameter) also decrease the activity: the presence of F, S, or O located at C8 as a part of the heterocycle located between the C8 and N1 positions (LEV, R-OFL, and RUF) is unfavorable for high cross-reactivity. This means that for Model 2, the character of the substituent at the C8 position may play a role.

## 4. Materials and Methods

### 4.1. Chemicals

All of the chemicals used in this investigation were of analytical grade. Danofloxacin (DAN), ofloxacin (OFL), levofloxacin (LEV), garenoxacin (GAR), pefloxacin (PEF), gatifloxacin (GAT), clinafloxacin (CLI), sarafloxacin (SAR), lomefloxacin (LOM), tosufloxacin (TOZ), sparfloxacin (SPA), difloxacin (DIF), pazufloxacin (PAZ), marbofloxacin (MAR), moxifloxacin (MOX), rufloxacin (RUF), norfloxacin (NOR), ciprofloxacin (CIP), enrofloxacin (ENR), pipemidic acid (PIP), nalidixic acid (NAL), oxolinic acid (OXO), orbifloxacin (ORB), enoxacin (ENO), nadifloxacin (NAD), flumequine (FLU), bovine serum albumin (BSA), ovalbumine (OVA), casein, 1-ethyl-3-(dimethylaminopropyl) carbodiimide hydrochloride (EDC), *N*-hydroxysuccinimide (NHS), *N*,*N*-dimethylformamide (DMF), ethylenediamine hydrochloride, triethylamine, sodium borohydride, glutardialdehyde, 4-aminomethylfluorescein (4-AMF), 3,3′,5,5′-tetramethylbenzidine (TMB), and Tween-20 were Sigma-Aldrich (St. Louis, MO, USA) products. Complete and incomplete Freund’s adjuvants were produced by Becton Dickinson (Franklin Lakes, NJ, USA). Peroxidase-labeled anti-rabbit immunoglobulins were from the Gamaleya Institute of Microbiology and Epidemiology (Moscow, Russia). All other chemicals (salts and solvents of analytical grade) were from Khimmed (Moscow, Russia).

### 4.2. Instrumentation

The microplate photometer EFOS 9305 made by Sapphire JSC MBP, Russia was used for photometric measurements in ELISA. The measurements were made with a wavelength of 450 nm. Fluorescence polarization was measured in photo-check mode by using the TDxFLx analyzer from Abbott Laboratories (Lake Bluff, IL, USA).

### 4.3. Experimental

#### 4.3.1. Synthesis of Cationized BSA (cBSA)

BSA carboxyl groups were modified using ethylenediamine, as described in [45]. The amount of 60 mg (0.88 μmol) of BSA was dissolved in 5 mL of distilled water, with the addition of a 0.5 mL solution containing 16.8 mg (88 μmol) EDC and 10.2 mg (88 μmol) NHS with vigorous stirring. This mixture was incubated for 15 min. After this, a solution of 13.0 mg (88 μmol) ethylenediamine hydrochloride was poured into the obtained preparation of the activated BSA, and 10 mL of 50 mM carbonate buffer pH 9.5 with 150 μL triethylamine was added. This mixture was incubated for 5 h with vigorous stirring. The suspension obtained with this technique was dialyzed against eight changes of distilled H_2_O and two changes of carbonate buffer for five days at 4 °C. The resulting solution was divided into aliquots and stored at −20 °C.

#### 4.3.2. Synthesis of Protein Conjugates and Fluorescein Tracer

The carboxyl group of the fluoroquinolone (CIP, CLI) was activated by using the carbodiimide method. Fluoroquinolone (14.7 µmol), 5.7 mg of EDC (30 µmol), and 3.5 mg NHS (30 µmol) were dissolved in 1 mL of DMF, and incubated with stirring for 2 h at room temperature. The protein (10 mg) was dissolved in 8 mL of carbonate buffer pH 9.5 with the addition of triethylamine (50 µL), and incubated for 1 h at 4 °C. The mixture containing fluoroquinolone with the activated carboxyl group was added slowly to the protein solution with constant stirring. The mixture obtained was incubated with stirring for 5 h at 25 °C in the dark. The removal of low-weight molecular compounds from the resulting conjugates was done by dialysis against distilled water for five days; on the last day, dialysis was performed against 0.01 M phosphate buffer pH 7.4. The dialyzed conjugates were frozen at −20 °C. As described above, CIP–BSA and CLI–cBSA conjugates were obtained.

The tracer PAZ-FITC was synthesized according to the methodology described by Mu et al. [41] with a few modifications. PAZ (2 mg) was dissolved in 0.5 mL of methanol. FITC (1.3 mg) and 25 µL of triethylamine were added with stirring. The solution was incubated for 24 h at room temperature. The tracer was separated by TLC by using chloroform/methanol/25% ammonium hydroxide (20:5:1, *v*/*v*/*v*) as the eluent. The resulting chromatogram was analyzed under UV light. The bands were scraped from the plate and extracted with the minimum sufficient volume of methanol, and stored in the dark at 4 °C.

The synthesis of CLI-C5-OVA conjugate was described previously [45] and is based on the use of glutardialdehyde as a crosslinking agent. OVA (0.11 μmol) and CLI (5.6 μmol) were dissolved in 8 mL of distilled water. An amount of 230 μL of 0.25% glutardialdehyde was added to the given mixture with vigorous stirring. The solution was incubated for 1 h at room temperature with constant vigorous stirring. Then, 500 μL of 0.22% sodium borohydride water solution was added and incubated for 30 min. The resulting conjugate was purified from low molecular weight substances by dialysis for five days (eight times against distilled water, with the last two times against phosphate buffer). The final preparation was divided into aliquots and stored at −20°C.

#### 4.3.3. Preparation of Antibodies and IgG Fraction

Male brush rabbits (*Sylvilagus bachmani*) at the age of three months were immunized every two weeks. The synthesized BSA and cBSA conjugates (see above) were mixed with Freund’s adjuvant before each immunization, to obtain fresh emissions. The conjugate (0.5 mg per 1.0 mL of 0.01 M phosphate buffer pH 7.4) and the adjuvant (complete one for the first immunization and incomplete one for the subsequent immunizations) were mixed at equal volume ratio (1:1). The emulsion was administered at 10–15 sites subcutaneously along the spine.

Blood sampling was carried out from the marginal ear vein, using Green Vac-Tube 0238 vacuum tubes with separating gel and a coagulation activator (SiO_2_). During the immunization, the serum was separated by centrifugation at 1000× *g* for 20 min, with IgG fraction separated via a 3-stage bedding method with 50%, 40%, and 33% ammonium sulfate, successively, at 4 °C. The obtained fraction of IgG was dissolved in 0.01 M phosphate buffer pH 7.4. The resulting solution was mixed with glycerol (1:1, *v*/*v*) and stored at −20 °C. Using the above described technique, two IgG preparations were obtained, namely CIP-113 and CLI-132.

#### 4.3.4. Enzyme-Linked Immunosorbent Assay (ELISA)

CLI-C5-OVA conjugate solutions in phosphate buffer solution were added to the wells of the polystyrene microplate at 100 μL/well. The plate was sealed using adhesive stickers and incubated for 20 h at + 4 °C. The wells were washed with distilled water (300 μL per well) twice, with the liquid carefully removed from the wells. The plates were dried at room temperature for two days in the dark. For ELISA, 50 μL of standard (analyte) and 50 μL of antibody solution were added to each well. The contents of the well were stirred and incubated for 1 h. The wells were washed three times with distilled water (300 μL per well). Horseradish peroxidase-labeled antibody solution was added (100 μL per well), and incubated at the same conditions for 30 min. The contents of the wells were removed and washed five times with distilled water (300 μL). The substrate mixture was added and incubated for 10–20 min. The reaction was terminated with the addition of 2 M H_2_SO_4_ (50 μL per well). The absorbency values were measured at a wavelength of 450 nm using a microplate photometer.

#### 4.3.5. Fluorescence Polarization Immunoassay (FPIA)

A solution of 50 μL of standard fluoroquinolone was mixed with 500 μL of tracer-diluted solution and 500 μL of the diluted antibody. A working solution of a tracer was added, and the fluorescence polarization signal was measured. To build the calibration curve, the experiment was repeated three times. The calibration curve was used to obtain the dependence between the relative polarization fluorescence and the logarithm of fluoroquinolone concentration.

Graphs on the dilution–signal coordinates were built using the obtained data, and the optimum graph was selected, based on the difference between the maximum and minimum signals to achieve the best resolution of the system. Using the calibration curve, the analytical parameters of the system were obtained, including IC_50_ (i.e., the fluoroquinolone concentration causing 50% decrease of the maximum polarization fluorescence signal).

#### 4.3.6. Cross-Reactivity

To characterize the cross-reactivity of the assay of compound A to the alternate compound B (CR_B_, %), the IC_50_ values for compounds A and B (IC_50,A_ and IC_50,B_, respectively) were determined, and the value was calculated as the measure of the assay cross-reactivity to alternate compound B:

CR_B_ = (IC_50,A_/IC_50,B_) × 100% (3)

### 4.4. QSAR Analysis

The activities of the studied quinolone compounds were presented as the logarithm of their cross-reactivities (Log CR). Quinolone molecules were divided into the training (80% of samples) and the test (20%) sets, using random number generation. The requirements for the maximum and minimum values in the test set were the following: (1) the maximum log CR value of the test set should be less than or equal to the maximum value of the log CR of the training set; (2) the minimum log CR value of the test set should be higher than or equal to the minimum value of the training set.

Linear regression equation has a form:

*y* = *a*_1_ × *x*_1_ + *a*_2_ × *x*_2_ +… + *a_n_* × *x_n_* + *c* (4)

where *y* is a dependent variable (log CR); *a*_1_, *a*_2_, and *a_n_* are regression coefficients for the corresponding *x*_1_, *x*_2_, and *x_n_* independent variables (descriptors), and *c* is a regression constant. A genetic function approximation algorithm [46] was applied to build linear regression equations with the help of free software developed in Jadavpur University (Kolkata, India) [47].

The best QSAR model was selected on the basis of the statistical parameters *r_tr_*^2^ (the coefficient of determination for the training set of compounds), *q*^2^ (the leave-one-out cross-validation coefficient), *LMO-q*^2^ (the leave-many-out cross-validation coefficient), the root mean square (RMS) error, the Friedman lack-of-fit error (LOF), and *pred_r*^2^ (predictive *r*^2^ for the test set of compounds). All QSAR models were validated and tested for their predictability, using a test set of five compounds.

#### 4.4.1. Conformational Analysis and Geometry Optimization

Conformational analysis and geometry optimization was performed by using Spartan’14 software [48]. The Generation of a series of low-energy conformers was done by using the molecular force field method (MMFF) [49]. Geometry optimization was done using the semi-empirical quantum-chemical method AM1 [50]. The most favorable low-energy conformer was chosen according to the total energy value, calculated using the Hartree–Fock quantum-chemical method with the 6-31G(d) basis set.

#### 4.4.2. Molecular Descriptors

Descriptors used in the QSAR study were the following: constitutional descriptors (molecular weight, H-acceptor and H-donor count, number of halogen atoms, number of rings, number of ring assemblies, number of chains, etc.), physicochemical descriptors (polarizability, logP, logD, water solubility, etc.), electrostatic descriptors (maximum positive charge, maximum negative charge, the number of atoms with positive charge, the number of atoms with negative charge, etc.), topological descriptors (Kappa shape indices, Kier & Hall molecular connectivity indices, Kier & Hall valence-modified connectivity indices, Zagreb index, etc.), 3D descriptors (Jurs descriptors, principal moments of inertia, shadow indices, volume, etc.) for a total of 238 descriptors. The descriptor values were obtained using both E-Dragon 1.0 [51] and Spartan’14 programs. Alignment was done before the calculation of 3D descriptors (Figure 2). Semi-empirical descriptors (HOMO energy, LUMO energy, dipole moment, polarizability, hydrophobicity, atom electrostatic charges, etc.) were obtained by using the AM1 method on the basis of AM1-optimized geometries.

The influence of a descriptor on a model was estimated according to the following equation:

(5) $$\alpha \left(x_{1}\right)=\frac{R^{2}\left(x_{1},x_{2},x_{3}\right)-R^{2}\left(x_{2},x_{3}\right)}{3\times R^{2}\left(x_{1},x_{2},x_{3}\right)-R^{2}\left(x_{1},x_{2}\right)-R^{2}\left(x_{1},x_{3}\right)-R^{2}\left(x_{2},x_{3}\right)}\times 100\%$$

where *α*(*x*_1_) is the relative contribution of the descriptor *x*_1_ to the model with three descriptors, $R^{2}\left(x_{1},x_{2},x_{3}\right)$ is the determination coefficient of the model with all three descriptors, and $R^{2}\left(x_{2},x_{3}\right)$ is the determination coefficient of the model with two descriptors *x*_2_ and *x*_3_.

Preprocessing of the independent variables was done by removing invariable constant descriptors, descriptors with too few nonzero values, and cross-correlated descriptors with correlation coefficients *r* > 0.5.

#### 4.4.3. Model Validation

The statistical parameter *adjusted r*^2^ (*r*^2^*adj*) was used to take into account the phenomenon of *r*^2^ increasing when extra variables are added to the model. While *r*^2^ is a measure of fit, adjusted *r*^2^ is instead a comparative measure of the suitability of alternative models. *R*^2^*adj* was calculated according to the following formula:

(5) $$r^{2}adj=1-\left(1-R^{2}\right)\frac{\left(N-1\right)}{\left(N-c\right)}\leq R^{2}$$

where *N* is a number of samples in the training set and *c* is a number of variables.

Friedman’s lack-of-fit error (*LOF*) [52] estimates the most appropriate number of descriptors, and it resists overfitting:

(6) $$LOF=\frac{SSE}{{\left(1-\frac{c+dp}{N}\right)}^{2}}$$

where *SSE* is the sum of squares of errors, *c* is a number of variables in a linear regression model, *d* is a user-defined smoothing parameter (was equal 0.5), *p* is the total number of descriptors contained in all model terms (except the constant term), and *N* is a number of samples in the training set.

Internal validation with the training set was carried out according to the leave-one-out (*q*^2^, LOO) method. To calculate the *q*^2^ parameter, each molecule in the training set was eliminated once, and the activity of the eliminated molecule was predicted by using the model developed by the remaining molecules. *Q*^2^ describes the internal stability of a model, and it was calculated by using the following equation:

(7) $$q^{2}=1-\frac{\sum {\left(y_{i}-{\hat{y}}_{i}\right)}^{2}}{\sum {\left(y_{i}-y_{mean}\right)}^{2}}$$

where *y_i_* and ${\hat{y}}_{i}$ are the actual and predicted log CR of the *i*th molecule in the training set, respectively, and *y_mean_* is the average activity of all molecules in the training set.

The leave-many-out (*LMO-q*^2^) method was also used for model validation. To calculate *LMO-q*^2^, four molecules of the training set (20% of samples contained in the training set) were eliminated once, and the activities of the four eliminated molecules were predicted by using the model developed by the remaining molecules. The equation used for the calculation of *LMO-q*^2^ is similar to one used for the calculation of *q*^2^.

For external validation, the activity of each molecule in the test set was predicted by using the model developed by the training set. *Pred_r*^2^ is indicative of the predictive power of the model and it was calculated as follows:

(8) $$pred\_r^{2}=1-\frac{\sum {\left(y_{act}-y_{pred}\right)}^{2}}{\sum {\left(y_{act}-y_{mean}\right)}^{2}}$$

where *y_act_* and *y_pred_* are the actual and predicted activities of the *i*th molecule in the test set, respectively, and *y_mean_* is the average activity of all molecules in the training set. Both summations are over all molecules in the test set.

## 5. Conclusions

In the present investigation, 26 quinolone molecules were evaluated for their cross-reactivities in two assay systems with different antibodies: anti-CIP and anti-CLI. Remarkable reactivity was found for CIP, CLI, DAN, GAR, and NAD in both systems. DIF and SAR were found to be the least active in this study. From an analysis of the results obtained, it is reasonable to conclude that recognition of the quinolone compounds by the anti-CIP and anti-CLI antibodies significantly depends on the presence of the cyclopropyl group at the N1 position, as well as the size of the molecule. Apparently, the importance of molecule size, shape, and cyclopropyl substituent is explained by steric effects and van der Waals interactions.

As demonstrated by statistical analysis, the QSAR models proposed in this study are useful, and they can be employed for recognizing different quinolone compounds. More QSAR research on the activity of quinolone residues in these and related systems is needed, which will serve as a guarantee of the further development of immunoassay methods for contaminant determination in animal-derived foods, primarily milk and dairy.

## Figures and Tables

**Table 1:** The cross-reactivity values of the fluoroquinolone compounds in the studied systems.

Immunogen		CIP-BSA	CLI-cBSA
	System	CIP-113/PAZ-FITC	CLI-132/CLI-С5-OVA, ELISA
Fluoroquinolone			
Ciprofloxacin (CIP)		100	73
Clinafloxacin (CLI)		52	100
Danofloxacin (DAN)		76	63
Difloxacin (DIF)		1	<1
Enoxacin (ENO)		39	5
Enrofloxacin (ENR)		57	33
Flumequine (FLU)		72	<1
Garenoxacin (GAR)		56	92
Gatifloxacin (GAT)		52	15
Levofloxacin (LEV)		-	<1
Lomefloxacin (LOM)		25	<1
Marbofloxacin (MAR)		19	<1
Moxifloxacin (MOX)		25	8
Nadifloxacin (NAD)		49	90
Nalidixic acid (NAL)		77	<1
Norfloxacin (NOR)		28	<1
Orbifloxacin (ORB)		58	10
Oxolinic acid (OXO)		36	<1
Pazufloxacin (PAZ)		24	<1
Pefloxacin (PEF)		26	<1
Pipemidic_acid (PIP)		44	<1
R-Ofloxacin (R-OFL)		-	<1
Rufloxacin (RUF)		13	<1
Sarafloxacin (SAR)		1	<1
Sparfloxacin (SPA)		43	10
Tosufloxacin (TOZ)		6	<1

**Table 2:** Molecular descriptor values for each molecule.

№	Name	N(>CH-)	S(>CH-)	N(Stereo)	Shadow-YZ
1	Ciprofloxacin (CIP)	1	0.187	0	40.61
2	Clinafloxacin (CLI)	2	0.031	1	45.23
3	Danofloxacin (DAN)	3	0.918	2	48.22
4	Difloxacin (DIF)	0	0	0	55.14
5	Enoxacin (ENO)	0	0	0	38.28
6	Enrofloxacin (ENR)	1	0.186	0	42.55
7	Flumequine (FLU)	1	0.096	1	35.64
8	Garenoxacin (GAR)	3	−2.935	1	48.49
9	Gatifloxacin (GAT)	2	0.29	1	47.77
10	Levofloxacin (LEV)	1	−0.158	1	42.71
11	Lomefloxacin (LOM)	1	0.074	1	43.80
12	Marbofloxacin (MAR)	0	0	0	42.93
13	Moxifloxacin (MOX)	3	0.887	2	47.48
14	Nadifloxacin (NAD)	2	−0.327	1	44.99
15	Nalidixic acid (NAL)	0	0	0	32.42
16	Norfloxacin (NOR)	0	0	0	38.77
17	Orbifloxacin (ORB)	3	−0.379	2	46.85
18	Oxolinic acid (OXO)	0	0	0	32.34
19	Pazufloxacin (PAZ)	1	−0.172	1	42.21
20	Pefloxacin (PEF)	0	0	0	39.69
21	Pipemidic_acid (PIP)	0	0	0	36.79
22	R-Ofloxacin (R-OFL)	1	−0.158	1	42.79
23	Rufloxacin (RUF)	0	0	0	41.23
24	Sarafloxacin (SAR)	0	0	0	54.61
25	Sparfloxacin (SPA)	3	−0.104	2	47.87
26	Tosufloxacin (TOZ)	1	−0.169	1	53.28

**Table 3:** Statistical results of QSAR models for fluoroquinolones obtained by multiple linear regression method.

№	Statistical Parameters	CIP-113/PAZ-FITC (Model 1)	CLI-132/CLI-С5-OVA, ELISA (Model 2)
1	*N*	19	20
2	*r_tr_* ^2^	0.803	0.934
3	*r* ^2^ *adj*	0.778	0.921
4	*q* ^2^	0.613	0.864
5	*LMO-q* ^2^	0.602	0.815
6	*pred_r* ^2^	0.944	0.640
7	*R* ^2^	0.866	0.885
8	*RMS error*	0.207	0.255
9	*LOF*	0.094	0.281

*N*: The number of samples in the training set; *r_tr_*^2^: the coefficient of determination for the training set; *r*^2^*adj*: *r*^2^ adjusted; *q*^2^: the leave-one-out cross-validation coefficient; *LMO-q*^2^: the leave-many-out cross-validation coefficient; *pred_r*^2^: the predictivity of the model toward the test set; *R*^2^: the coefficient of determination for both the training set and the test set; *RMS error*: the root mean square error; *LOF*: the Friedman lack-of-fit error.

## Supplementary Materials

Supplementary materials are available online at http://www.mdpi.com/1422-0067/20/2/265/s1. Table S1: 3D geometry of the most favorable low-energy conformer for each molecule optimized with AM1 method; experimental and predicted cross-reactivity values.

## Abbreviations

BSA	Bovine serum albumin
CIP	Ciprofloxacin
CLI	Clinafloxacin
CR	Cross-reactivity
FQs	Fluoroquinolones
OVA	Ovalbumin
PAZ	Pazufloxacin
QSAR	Quantitative Structure-Activity Relationship
