# Supplementary material for: Ciprofloxacin and Clinafloxacin Antibodies for an Immunoassay of Quinolones: Quantitative Structure–Activity Analysis of Cross-Reactivities
Source: Int J Mol Sci. 2019 Jan 11;20(2):265. doi: 10.3390/ijms20020265 (PMC6359390; doi:10.3390/ijms20020265)
Supplement: Supplementary file 1 [file ijms-20-00265-s001.pdf]

Article

Ciprofloxacin and clinafloxacin antibodies for an immunoassay of quinolones: quantitative structure-activity analysis of cross-reactivities

Andrey A. Buglak[1,2,*], Ilya A. Shanin[3,4], Sergei A. Eremin[3], Hong-Tao Lei[5], Xiangmei Li[5], Anatoly V. Zherdev[1], Boris B. Dzantiev[1]

[1] A. N. Bach Institute of Biochemistry, Research Center of Biotechnology of the Russian Academy of Sciences, 33 Leninsky Prospect, Moscow 119071, Russia

[2] St. Petersburg State University, 7/9 Universitetskaya nab., St. Petersburg 199034 Russia

[3] Chemical Department, M. V. Lomonosov Moscow State University, Leninskie Gory, Moscow 119991, Russia

[4] XEMA Company Limited, Ninth Parkovaya street 48, Moscow 105264, Russia

[5] Guangdong Provincial Key Laboratory of Food Quality and Safety, South China Agricultural University Guangzhou 510642, China

* Correspondence: andreybuglak@gmail.com; Tel.: +7 (495) 954-27-32

Supplementary Information

Table S1. 3D geometry of the most favorable low-energy conformer for each molecule optimized with AM1 method (carbon atoms are colored in grey, nitrogens – blue, oxygens – red, fluorines – yellow, hydrogens - white); experimental and predicted cross-reactivity values.

№	Compound	CIP-113 / PAZ-FITC		CLI-132 / CLI-C5-OVA, ELISA	
		**Exp.**	**Pred.**	**Exp.**	**Pred.**
1	Garenoxacin 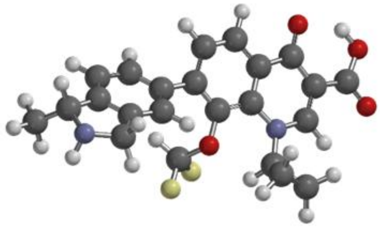	1.75	1.69	1.96	2.04
2	Gatifloxacin [T1] 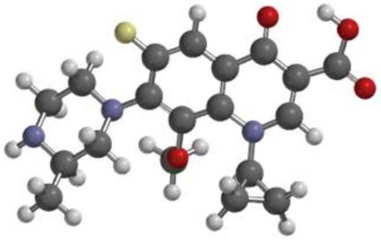	1.72	1.41	1.18	1.38
3	Danofloxacin 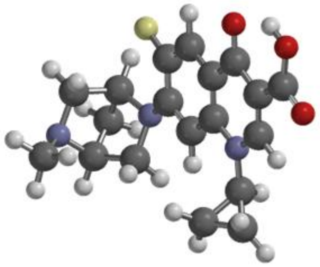	1.88	1.71	1.80	1.45
4	Difloxacin [T1] 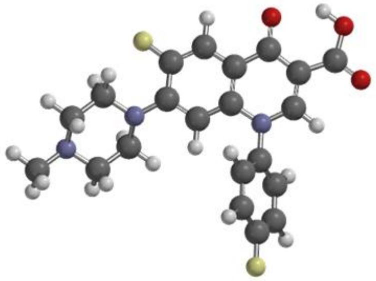	0	0.18	-0.30	-0.22
5	Clinafloxacin	1.72	1.60	2.0	1.30

	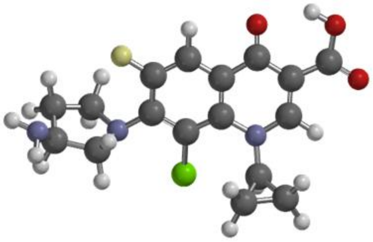				
6	Levofloxacin 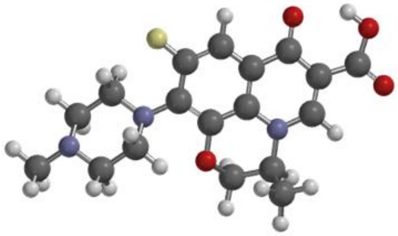	-	-	-0.30	-0.39
7	Lomefloxacin 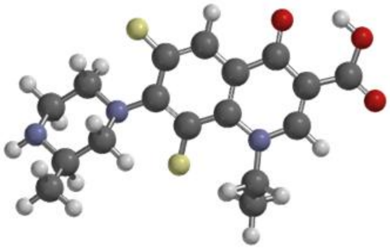	1.40	1.37	-0.30	-0.32
8	Marbofloxacin 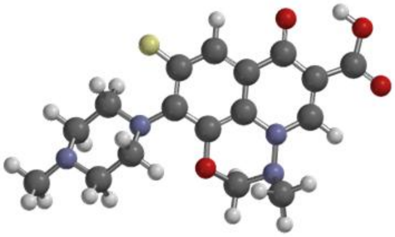	1.28	1.10	-0.30	-0.22
9	Moxifloxacin 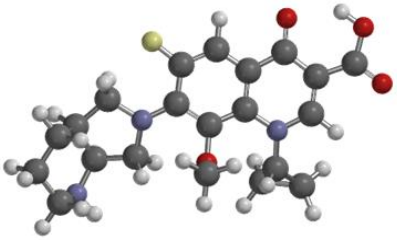	1.40	1.76	0.90	1.45

10	Nadifloxacin[T2] 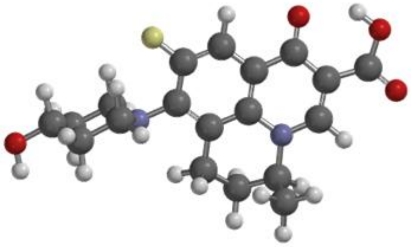	1.69	1.62	1.95	1.19
11	Nalidixic acid[T2] 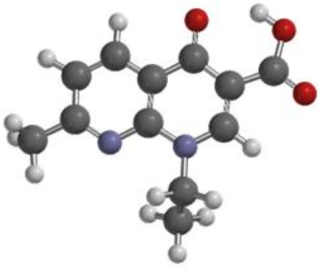	1.89	1.89	-0.30	-0.22
12	Norfloxacin 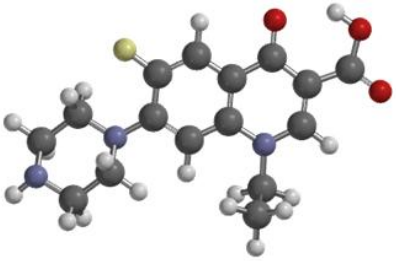	1.45	1.41	-0.30	-0.22
13	Orbifloxacin 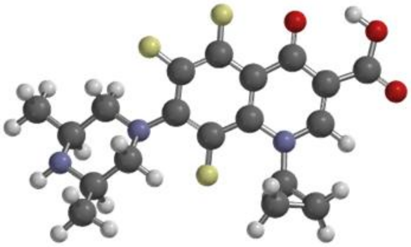	1.76	1.81	1.0	1.06
14	Oxolinic acid 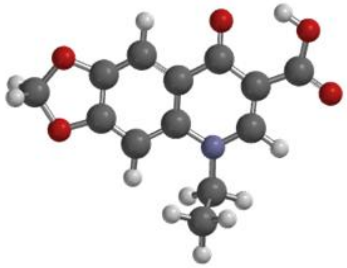	1.56	1.90	-0.30	-0.22

15	R-Ofloxacin 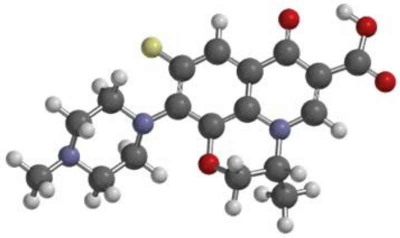	-	-	-0.30	-0.39
16	Pazufloxacin 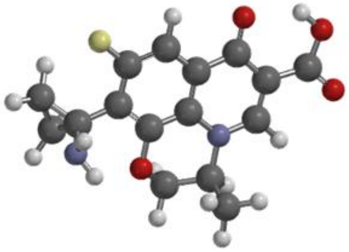	1.38	1.49	-0.30	-0.39
17	Pefloxacin [T1, T2] 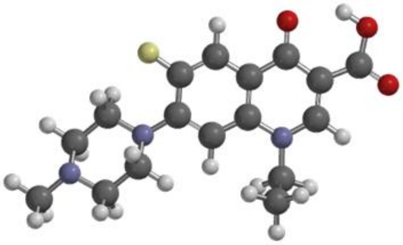	1.41	1.34	-0.22	-0.30
18	Pipemidic_acid 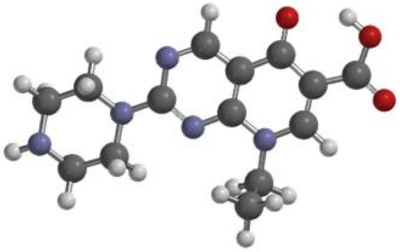	1.64	1.56	-0.30	-0.22
19	Rufloxacin [T2] 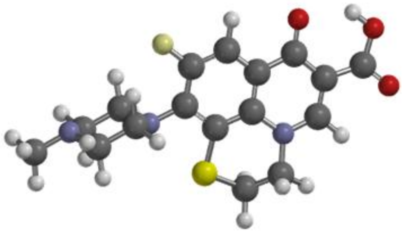	1.11	1.23	-0.22	-0.30

20	Sarafloxacin 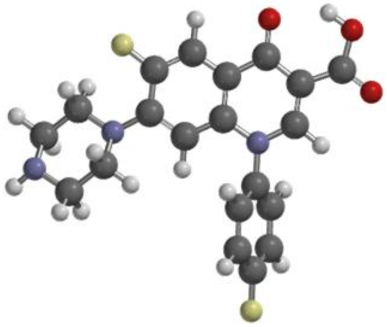	0	0.22	-0.30	-0.22
21	Sparfloxacin 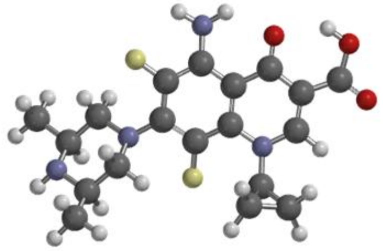	1.63	1.74	1.0	1.14
22	Tosufloxacin [T1] 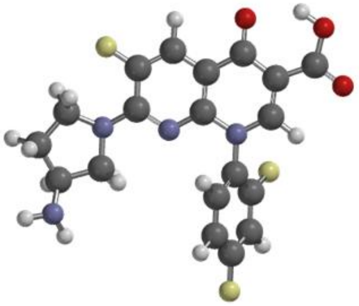	0.78	0.65	-0.30	-0.39
23	Flumequine 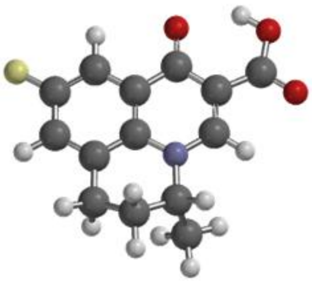	1.86	1.99	-0.30	-0.32

24	Ciprofloxacin 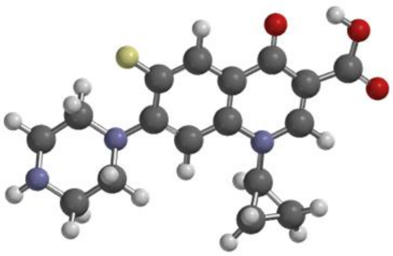	2.0	1.61	1.86	1.47
25	Enoxacin [T1, T2] 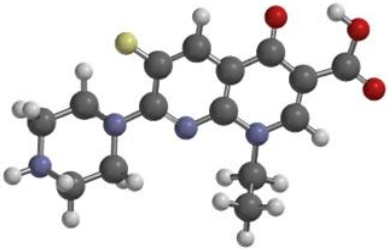	1.59	1.45	0.70	-0.22
26	Enrofloxacin 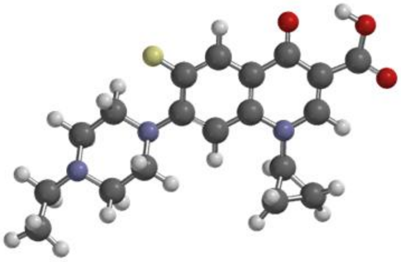	1.76	1.46	1.52	1.46

[T] – Compounds presented in the test set
